# UBAP2 negatively regulates the invasion of hepatocellular carcinoma cell by ubiquitinating and degradating Annexin A2

**DOI:** 10.18632/oncotarget.8783

**Published:** 2016-04-18

**Authors:** Dou-Sheng Bai, Chao Wu, Liu-Xiao Yang, Chi Zhang, Peng-Fei Zhang, Yi-Zhou He, Jia-Bin Cai, Zheng-Ji Song, Zhao-Ru Dong, Xiao-Yong Huang, Ai-Wu Ke, Guo-Ming Shi

**Affiliations:** ^1^ Department of Hepatobiliary and Pancreatic Surgery, Clinical Medical College of Yangzhou University, Jiangsu, 225001, P.R. China; ^2^ Liver Cancer Institute and Department of Liver Surgery of Zhongshan Hospital, Fudan University, Key Laboratory of Carcinogenesis and Cancer Invasion (Fudan University), Ministry of Education, Shanghai, 200032, P.R. China; ^3^ Department of Digestion, The First People's Hospital of Yunnan Province, Yunnan, 650000, P.R. China

**Keywords:** hepatocellular carcinoma, ubiquitin associated protein 2, Annexin A2, ubiquitination, invasion

## Abstract

The ubiquitin-dependent proteasomal degradation of proteins controls signaling and cellular survival. In this study, we found that ubiquitin associated protein 2 (UBAP2) was significantly downregulated in hepatocellular carcinoma (HCC) tissues compared with adjacent normal tissues. Furthermore, higher expression of UBAP2 in cancer tissues was correlated with good prognosis in HCC patients. Knockdown of UBAP2 significantly enhanced the invasion and proliferation of HCC cells *in vitro* and promoted tumor growth *in vivo*, while enforced expression of UBAP2 impaired the aggressive ability and tumor growth of HCC cells. Mechanistically, UBAP2 formed a complex with Annexin A2 and promoted the degradation of Annexin A2 protein by ubiquitination, and then inhibited HCC progression. Collectively, UBAP2 appears as a novel marker for predicting prognosis and a therapeutic target for HCC.

## INTRODUCTION

Hepatocellular carcinoma (HCC) ranks as the fifth and seventh most common cancer in men and women, respectively, and the third most common cause of cancer-related mortality worldwide [1]. This disease is characterized by highly recurrent rate after curative resection and resistance to chemotherapy [2, 3]. Therefore, it is imperative to have a better understanding of the key genes and their mechanisms related to liver tumorigenesis and progression, and thus develop new diagnostic and therapeutic strategy. The function of these oncogenes and tumor suppress genes mainly depend on the level of their proteins influenced by post-translational modifications such as ubiquitination, phosphorylation and acetylation [4]. However, the mechanism of post-translational regulation of these genes in HCC remains to be largely uncovered.

The ubiquitin-proteasome pathway (UPP) is a common cellular process for protein degradation in eukaryotes and involves in the regulation of cellular process including cell cycle, transcription, apoptosis, cell adhesion, angiogenesis, and tumor growth [5]. Abnormal UPP usually results in many diseases, particularly cancer [6]. Recently, the UPP have emerged as important therapeutic targets in prostate cancer and HCC [7, 8]. Ubiquitin associated protein 2 (UBAP2) contains a ubiquitin associated (UBA) domain and damages the structure and function of target proteins [9, 10]. However, its function in HCC is still unknown.

In the present study, we attempted to illustrate the role and relevant molecular mechanism of UBAP2 in the invasion of HCC cells. The clinical significance of UBAP2 and its interacting protein in HCC patients were also investigated.

## RESULTS

### UBAP2 weakly expresses in tumor tissues and intensity of UBAP2 inversely correlates with prognosis in HCC patients

**Figure 1:**
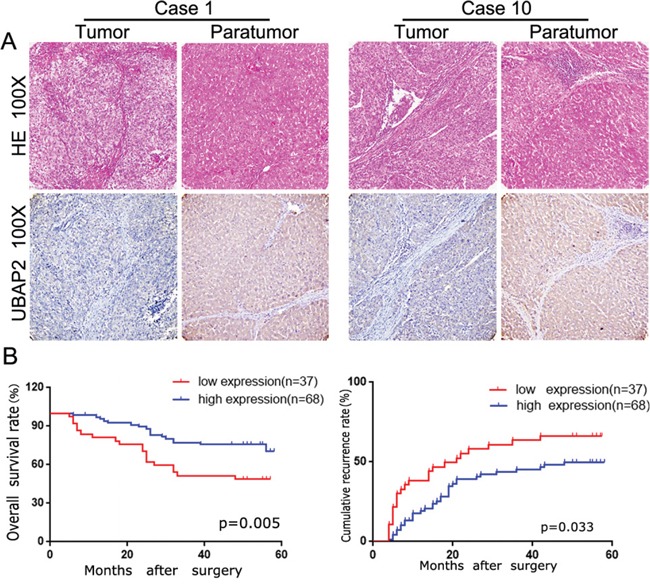
The UBAP2 expression and its clinical significance in HCC patients **A.** After identification by H&E, representative tumor tissue and corresponding paratumoral tissues in HCC patients were stained for UBAP2 by IHC. **B.** the role of UBAP2 expression in overall survival and cumulative recurrence rate of 105 HCC patients was investigated.

Firstly, we used to IHC staining to investigate the expression of UBAP2 in 105 HCC tissues. As shown in Figure 1A, the positive staining for UBAP2 was localized in the cytoplasm of liver cells and tumor cells. The intensity of UBAP2 expression in HCC tissues was weaker than that in adjacent normal tissues. In tumor tissues, UBAP2 expression varied and was strong in 34 (32.4%), moderate in 34 (67.7%), weak in 22 (21.0%), and negative in 15 (14.3%) case. We then assayed the relationship between UBAP2 expression and the clinic-pathological characteristics. We dichotomized 105 patients into UBAP2-high (strong and moderate; n = 68) and UBAP2-low (negative and weak; n = 37) groups. Our results revealed that UBAP2-high subgroups had smaller tumor size (Table 1). Importantly, survival analysis showed that UBAP2-low groups had poorer prognosis in term of overall survival (OS; p = 0.005) and cumulative recurrence rate (p = 0.033) (Figure 1B). Multivariate analysis showed that UBAP2 intensity in cancer tissues was an independent prognosticator for OS (Table 2 and 3). Above data indicate that UBAP2 expression promote the tumor progression of HCCs.

**Table 1:** Correlation between UBAP2 and clinicopathological features in 105 hepatocellular carcinoma patients

Variables		UBAP2 staining	p value
	High expression	Low expression	
Sex			
Male	61	32	0.620
Female	7	5	
Age (years)			
≥53	31	18	0.764
<53	37	19	
HBsAg			
Positive	55	25	0.126
Negative	13	12	
Child-Pugh classification*			
A	67	36	0.666
B	1	1	
ALT (U/ml)*			
≥75	9	3	0.430
<75	59	34	
Serum AFP (ng/ml)			
≥20	39	24	0.453
<20	29	13	
Liver cirrhosis*			
Yes	62	33	0.740
No	6	4	
Tumor diameter (cm)			
≥5	18	21	0.002
<5	50	16	
Tumor number*			
Multiple	11	4	0.453
Single	57	33	
Microvascular invasion			
Yes	12	12	0.085
No	56	25	
Tumor encapsulation			
Yes	33	13	0.186
None	35	24	
Tumor differentiation			
III/IV	21	10	0.679
I/II	47	27	
TNM stage			
III/IV	52	23	0.121
I/II	16	14	

Abbreviations: HCC, hepatocellular carcinoma; HBsAg, hepatitis B surface antigen; ALT, alanine transaminase; AFP, alpha-fetoprotein; TNM, tumor node metastasis.

* Fisher's Exact Test

**Table 2:** Univariate and multivariate analyses of factors associated with overall survival

Factors	Univariate, p	Mulvariate		
		HR	95%Cl	p value
Sex (female vs. male)	0.231			NA
Age (years) (≥53 vs. <53)	0.736			NA
HBsAg (positive vs. negative)	0.643			NA
Child-Pugh classification (A vs. B)	0.531			NA
Liver cirrhosis (yes vs. no)	0.327			NA
Serum AFP, ng/mL (≥20 vs. <20)	0.576			NA
Serum ALT, U/L (≥75 vs. <75)	0.634			NA
Tumor size (diameter, cm) (≥5 vs. <5)	0.141			NA
Tumor number (multiple vs. single)	0.272			NA
Tumor differentiation (III/IV vs. I/II.)	0.592			NA
Tumor encapsulation (yes vs. no)	0.085			NA
Microvascular invasion (yes vs. no)	0.045			NS
TNM stage (I/II vs. III/IV)	0.018			NA
UBAP2 expression (high vs. low)	0.007	0.428	0.221-0.829	0.012

Abbreviations: 95% CI, 95% confidence interval; AFP, alpha-fetoprotein; TNM, tumor node metastasis; HBsAg, hepatitis B surface antigen; HR, hazard ratio; NA, not adopted; NS, not significant.

Cox proportional hazards regression model.

**Table 3:** Univariate and multivariate analyses of factors associated with cumulative recurrence

Factors	Univariate, p	Mulvariate		
		HR	95%Cl	p value
Sex (female vs. male)	0.772			NA
Age (years) (≥53 vs. <53)	0.600			NA
HBsAg (positive vs. negative)	0.388			NA
Child-Pugh classification (A vs. B)	0.395			NA
Liver cirrhosis (yes vs. no)	0.085			NA
Serum AFP, ng/mL (≥20 vs. <20)	0.093			NA
Serum ALT, U/L (≥75 vs. <75)	0.346			NA
Tumor size (diameter, cm) (≥5 vs. <5)	0.163			NA
Tumor number (multiple vs. single)	0.134			NA
Tumor differentiation (III/IV vs. I/II.)	0.985			NA
Tumor encapsulation (yes vs. no)	0.103			NA
Microvascular invasion (yes vs. no)	0.048			NS
TNM stage (I/II vs. III/IV)	0.091			NA
UBAP2 expression (high vs. low)	0.038			NS

Abbreviations: 95% CI, 95% confidence interval; AFP, alpha-fetoprotein;TNM, tumor node metastasis; HBsAg, hepatitis B surface antigen; HR, hazard ratio; NA, not adopted; NS, not significant.

Cox proportional hazards regression model.

### Upregulation of UBAP2 expression inhibits the invasion and proliferation of HCC cells *in vitro* and blocks tumor growth *in vivo*

**Figure 2:**
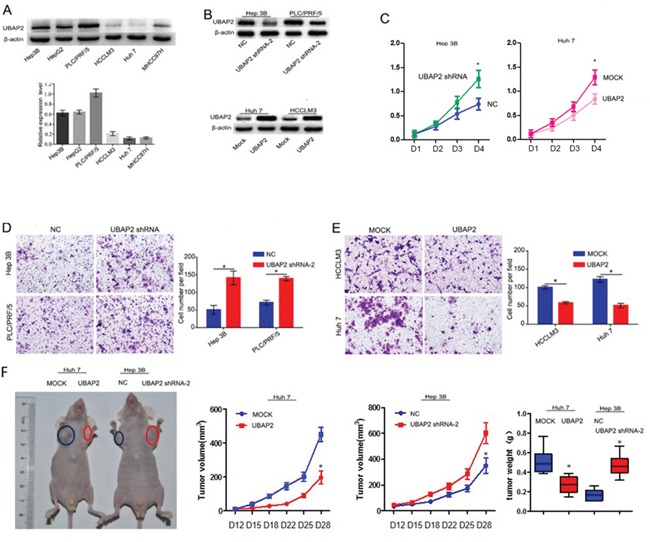
Role of UBAP2 expression in the progression of HCC cells **A.** UBAP2 expression in 6 HCC cell lines was examined by western blot and qRT-PCR. **B.** stable UBAP2 expression in Hep3B, PLC/PRF/5, Huh 7 and HCCLM3 cells were constructed and validated by western blot. **C.** The cell proliferation of Huh 7 and Hep 3B transfectants *in vitro* was examined by MTT assay. *p <0.05. **D, E.** The invasive ability of Huh 7, HCCLM3, Hep 3B, and PLC/PRF/5 transfectants *in vitro* was examined by invasion assay. *p <0.05. **F.** Tumor growth was assayed in HCC cells with enforced expression of UBAP2 (Huh 7-UBAP2) or HCC cells with inhibited expression of UBAP2 (Hep3B-shRNA-UBAP2) by a SC xenograft tumor model.

To further test the role of UBAP2 expression in the progression of HCC cells, we analyzed the expression of UBAP2 in 6 HCC cell lines (Figure 2A). The results showed that high metastatic HCCLM3 and Huh 7 cells expressed weak UBAP2, while low metastatic Hep3B and PLC/PRF/5 cells had high level expression of UBAP2. Then, we used shRNA interference to reduce UBAP2 expression in Hep3B and PLC/PRF/5 cells, and transfected lentiviral vector-mediated UBAP2 cDNA to increase UBAP2 expression in the HCCLM3 and Huh 7 cells. Western blot analysis exhibited stable expression of UBAP2 protein in target cells (Figure 2B). Next, we examined the role of UBAP2 expression in the proliferation and invasion of HCC cells. The results showed that inhibition of UBAP2 expression significantly increased the cell proliferation and invasive abilities of Hep3B and PLC/PRF/5 cells, while upregulation of UBAP2 expression significantly reduced the cell proliferation and invasiveness of HCC cells (Figure 2C–2E). Then, we used a SC xenograft tumor model to assay the role of UBAP2 in tumor growth. Our results revealed that down-regulation of UBAP2 expression significantly promoted tumor growth *in vivo* (Figure 2F). These results suggest that downregulation of UBAP2 expression promote the progression of HCC *in vitro* and tumor growth *in vivo*.

### UBAP2 forms a complex with Annexin A2 and promotes Annexin A2 degradation by ubiquitination

**Figure 3:**
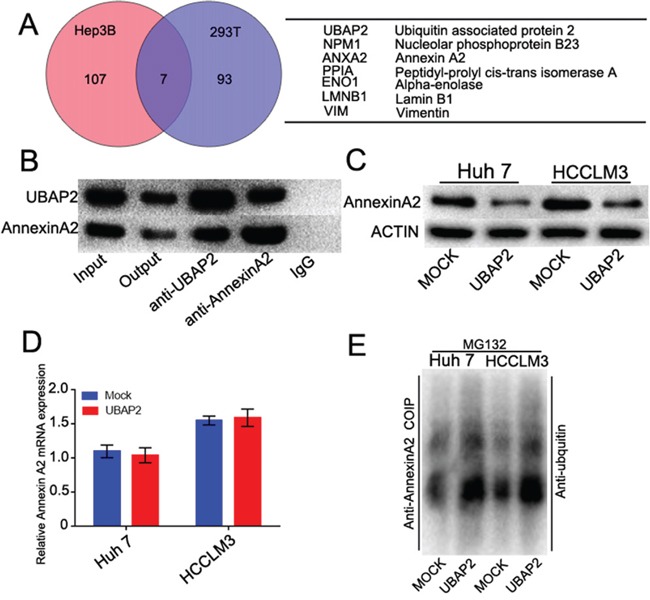
UBAP2 formed a complex with Annexin A2 and promoted Annexin A2 degradation **A.** Identification of binding partners of UBAP2 by combination of co-IP and 2D-LS/MS in Hep3B and 293T cells. Venn diagram showed the number of binding partners of UBAP2. Seven overlapped proteins were listed in the Table. **B.** Co-IP analysis was used to validate the formation of UBAP2/Annexin A2 complex in PLC/PRF/5 cells. **C.** Western blot showed that enforced UBAP2 expression down-regulation the protein levels of Annexin A2. **D.** qRT-PCR showed that UBAP2 had no influence in the Annexin A2 mRNA. **E.** Ubiquitination assay showed that Annexin A2 degradation was promoted by UBAP2 overexpression.

To determine the mechanism of the role of UBAP2 in the progression of HCCs, a combination of co-IP with MS was used to identify the interactome of UBAP2 in Hep3B and 293T cells. Among the two sets of proteins, 7 overlapped proteins were found, including Annexin A2, nucleolar phosphoprotein B23, peptidyl-prolyl cis-transisomerase A, alpha-enolase, vimentin and lamin B1 (Figure 3A). Given the role of Annexin A2 in regulation of cancer development [17], we here focus on the relationship between Annexin A2 and UBAP2. Reciprocal co-IP assay revealed that UBAP2 formed a complex with Annexin A2 in PLC/PRF/5 cells (Figure 3B). Importantly, enforced UBAP2 expression in the Huh 7 and HCCLM3 cells resulted in a correspondingly decreased expression of Annexin A2 protein (Figure 3C). However, UBAP2 expression did not influence in Annexin A2 mRNA (Figure 3D). Recent papers have testified the role of UBAP2 in the protein degradation [9]. We then investigated that the role of UBAP2 in the degradation of Annexin A2 protein by ubiquitination. Ubiquitination assay showed that the expression of Annexin A2 in Huh 7-UBAP2 cells and HCCLM3-UBAP2 cells was much stronger than that in corresponding controls (Figure 3E). About results indicate that upregulation of UBAP2 expression probably promote the ubiquitination and degradation of Annexin A2 protein.

### Enforced Annexin A2 expression turnovers the inhibited invasion induced by overexpression of UBAP2

**Figure 4:**
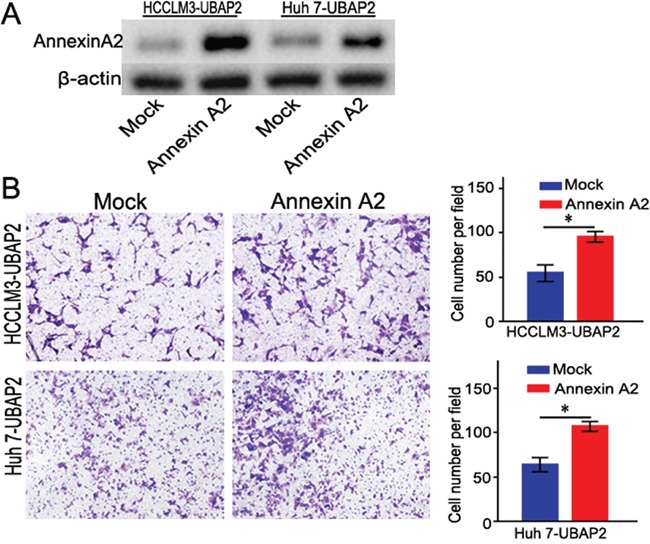
Enforced Annexin A2 expression rescued the invasive ability of HCC cells induced by UBAP2 **A.** Stably transfected HCC cells with overexpression of Annexin A2 were validated by western blot. **B.** Overexpression of Annexin A2 could rescue the invasive ability of HCC cells induced by UBAP2. *p <0.05

To further confirm that the role of Annexin A2 in UBAP2-mediated invasion of HCC cells, we transfected Annexin A2 cDNA into HCC cells with high level of UBAP2 expression to enhance Annexin A2 expression (Figure 4A). Interestingly, the invasive ability of HCCLM3-UBAP2 and Huh 7-UBAP2 cells was rescued after these cells were transfected by Annexin A2 cDNA(Figure 4B).

### High level of Annexin A2 expression correlates with poor prognosis of HCC patients

**Figure 5:**
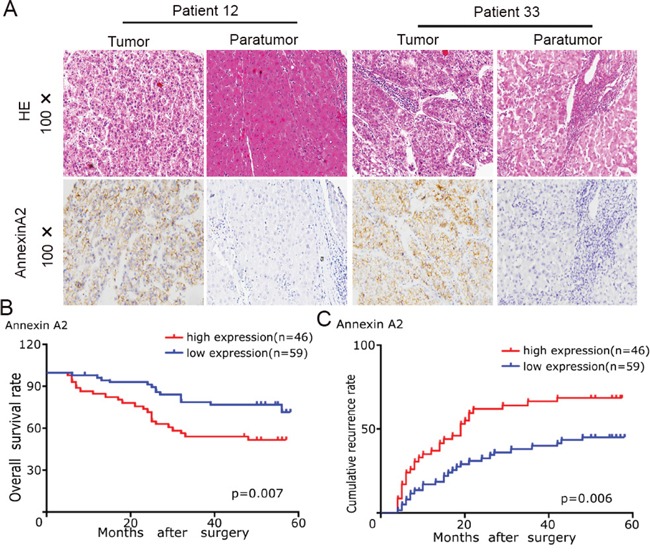
The Annexin A2 expression and its clinical significance in HCC patients **A.** Representative tumor tissues in HCC patients were stained for Annexin A2 by IHC. **B, C**. the role of Annexin A2 expression in overall survival and recurrence of 105 HCC patients was investigated.

We investigated the clinical significance of Annexin A2 expression in the same cohort of HCC patients. IHC revealed that positive staining for Annexin A2 expression was localized in cell membrane and cytoplasm of tumor cells (Figure 5A). The intensity of Annexin A2 expression in tumor tissue was stronger than that in matched adjacent liver tissue (Figure 5A). According to the intensity of Annexin A2 expression, we also classified 105 patients into Annexin A2-high (n = 46) and Annexin A2-low (n = 59) groups. We analyzed the correlation between Annexin A2 expression and clinicopathological features. Our results revealed that Annexin A2-high subgroup had larger tumor size (p = 0.016, supplementary Table 1). Survival analysis revealed that Annexin A2-high groups had poorer prognosis (Figure 5B and 5C). Multivariate analysis showed that Annexin A2 expression in tumor tissues was an independent prognosticator for OS and RFS (supplementary Table 2 and 3). Finally, we assayed the correlation between UBAP2 and Annexin A2 in tumor tissues. Semi-quantitative analysis for IHC showed that the Annexin A2 expression was negative related to the expression of UBAP2 (R = −0.634, p < 0.001, supplementary Table 4). These data suggest that Annexin A2 involve in the progression of HCC through formation of complex with UBAP2.

## DISCUSSION

Here, we demonstrated that UBAP2 weakly expressed in tumor samples and its expression inversely correlated with prognosis of HCC patients. Moreover, enforced UBAP2 expression in HCC cells could impair the invasive ability *in vitro* and inhibited tumor growth *in vivo*. To our knowledge, it is the first time to report that UBAP2 inhibit the progress of HCC.

Recent studies reported the contradictory roles of abnormal UPP in different cancers. Dolcet et al. reported that inhibition of the UPP could activate NF-κB and induce cell death of endometrial carcinoma [11]. Similarly, UPP inhibitor could inhibit the invasiveness of leukemic cells [12] and Burkitt's lymphoma [13] through inducement of apoptosis. Contrarily, Gobbi G et al showed that inhibition of UPP could downregulate the expression of low-molecular-weight proteins (LMP)-2 and -10, and promote the metastasis in breast cancer [14]. Thus, whether UPP acts as an oncogene or tumor suppressor gene mainly depends on the context. In present study, our evidences support that UBAP2 appears as a tumor suppressor gene in the HCCs.

Another interesting result from the our study is that UBAP2 forms a complex with Annexin A2 and promote the degradation of Annexin A2 protein through ubiquitination. In present study, we used the combination of co-IP and MS to identify 6 molecular partners of UBAP2, including Annexin A2. Accumulating evidence have showed that Annexin A2 is aberrantly expressed in a wide spectrum of cancers, including HCC [15-17]. Annexin A2 involved in several pathological processes, such as tumor cell adhesion, proliferation, apoptosis, tumor neoangiogenesis, invasion and metastasis [15, 16]. In HCCs, Annexin A2 could activate with HAb18G/CD147. Downregulation of Annexin A2 significantly decreased the secretion of matrix metalloproteinases (MMPs), migration ability, and invasive potential [18]. Annexin A2 could also inhibit the trafficking of CD147-harboring membrane microvesicles and enhance the migration and invasion potential of tumor cells [19]. In addition, Annexin A2 may downregulate β-catenin and cyclin D1 expression and involve in the inhibition of cell cycle [20]. Our results showed that enforced expression UBAP2 decreased the expression of Annexin A2 in HCC cells. Importantly, upregulation of UBAP2 expression could promote the ubiquitination and degradation of Annexin A2 protein. Even more importantly, transfection of Annexin A2 into Huh 7-UBAP2 cells and HCCLM3-UBAP2 cells could rescue the invasive ability of HCC cells. These data support the notion that upregulation of UBAP2 forms a complex with Annexin A2, and promotes its degradation by ubiquitination, thus inhibits the progression of HCCs. Certainly, our study have some limitations. For example, the role of other molecular partners in the UBAP2-mediated invasion remains to be addressed in the future.

In summary, we demonstrate that overexpression of UBAP2 promotes ubiquitination and the degradation of Annexin A2, thus impairs the progression of HCC, indicating that UBAP2 appear as a novel marker for predicting prognosis and a therapeutic target for HCC.

## MATERIALS AND METHODS

### Patients, follow-up, and treatment modality

A total of 105 pathologically confirmed HCC patients, who underwent curative resection at Liver Cancer Institute of Zhongshan Hospital, Fudan University between January 2006 and December 2007, were enrolled in this study. The inclusion criteria, treatments and follow-up were described previously [21].

### Construction of tissue microarrays (TMA) and immunohistochemistry (IHC)

TMA were constructed as described in our earlier study [22]. IHC staining was done as previously described [22].

### Cell culture

The human HCC cell lines HepG2, Hep3B, Huh 7, MHCC97H, PLC/PRF/5 and HCCLM3 were used in this study. These cell lines were maintained routinely [22].

### Lentivirus production and transduction of target cells

The UBAP2 lentiviral vector and UBAP2 shRNA expression lentivirus were constructed (Shanghai GeneChemCo.) and transfected as described elsewhere [23, 24]. The target sequences of the shRNAs were listed in Supplementary Table 5.

### Cell proliferation and invasion assay

Cell proliferation was performed as previously described [22]. Cell invasion was measured by a transwell matrigel assay as previously described with minor revision [25, 26]. HCC cells were seeded into the filter without pre-coated Matrigel and incubated 36h for Hep3B, PLC/PRF/5 and HCCLM3 cells and 24 h for Huh-7 cells.

### Western blot and quantitative real-time polymerase chain reaction (qRT-PCR)

Western blot and qRT-PCR were performed as described previously [21]. Rabbit anti-human UBAP2 antibody (Abcam, Cambridge, MA, USA) and Rabbit anti-human Annexin A2 antibody (Abcam, Cambridge, MA, USA) were used as primary antibody. qRT-PCR was performed to evaluate the expression level of Annexin and β-actin was used as an endogenous control. The primers used were Annexin A2, forward, 5′-TGACGCTGGAGTGAAGAGGAA-3′ and reverse, 5′-GCCCTTAGTGTCTTGCTGGATA-3: β-actin, forward, 5′-GTGGACATCCGCAAAGAC-3′ and reverse, 5′-AAAGGG TGTAACGCAACTA-3′. All experiments were performed in triplicate.

### Co-immunoprecipitation (Co-IP), two-dimensional liquid chromatograph tandem mass spectrometry (2D-LC-MS/MS) and ubiquitination assay

Combination of co-IP with MS were used to identify the binding partners of UBAP2 in 293T and Hep3B cells as described in our earlier study [21]. Ubiquitination assay was used to investigate the ubiquitinated role of UBAP2 as described previously [22].

### *In vivo* tumor growth assay

Subcutaneous (SC) xenograft tumor models were established as previous described [22]. Tumor growth was assayed as previous described [22].

### Statistical analysis

Statistical analysis was performed using the SPSS 19.0 software (Chicago, IL, USA). All tests were two-tailed and p < 0.05 was considered statistically significant.

## SUPPLEMENTARY TABLES



## Abbreviations

UBAP2: ubiquitin associated protein 2
HCC: hepatocellular carcinoma
OS: overall survival
RFS: recurrence-free survival
DMEM: Dulbecco's modified Eagle medium
qRT-PCR: quantitative real-time polymerase chain reaction
shRNA: short hairpin RNA
Co-IP: Co-immunoprecipitation
2D-LC-MS/MS: two-dimensional liquid chromatograph tandem mass spectrometry
TMA: tissue microarray
UPP: ubiquitin-proteasome pathway.
