# Supplementary material for: UBAP2 negatively regulates the invasion of hepatocellular carcinoma cell by ubiquitinating and degradating Annexin A2
Source: Oncotarget. 2016 Apr 18;7(22):32946–55. doi: 10.18632/oncotarget.8783 (PMC5078065; doi:10.18632/oncotarget.8783)
Supplement: Supplementary file 1 [file oncotarget-07-32946-s001.pdf]

## SUPPLEMENTARY TABLES

**Supplementary Table S1: Correlation between Annexin A2 and clinicopathological features in 105 hepatocellular carcinoma patients**

| Variables | Annexin A2 staining | | |
|---|---|---|---|
| | High expression | Low expression | p value |
| Sex* | | | |
| Male | 42 | 51 | 0.437 |
| Female | 4 | 8 | |
| Age (years) | | | |
| ≥53 | 25 | 24 | 0.164 |
| <53 | 21 | 35 | |
| HBsAg | | | |
| Positive | 32 | 48 | 0.159 |
| Negative | 14 | 11 | |
| Child-Pugh classification* | | | |
| A | 45 | 58 | 0.859 |
| B | 1 | 1 | |
| ALT (U/ml) | | | |
| ≥75 | 5 | 7 | 0.874 |
| <75 | 41 | 52 | |
| Serum AFP (ng/ml) | | | |
| ≥20 | 27 | 36 | 0.810 |
| <20 | 19 | 23 | |
| Liver cirrhosis | | | |
| Yes | 41 | 54 | 0.678 |
| No | 5 | 5 | |
| Tumor diameter (cm) | | | |
| ≥5 | 23 | 16 | 0.016 |
| <5 | 23 | 43 | |
| Tumor number | | | |
| Multiple | 5 | 10 | 0.377 |
| Single | 41 | 49 | |
| Microvascular invasion | | | |
| Yes | 14 | 10 | 0.103 |
| No | 32 | 49 | |
| Tumor encapsulation | | | |
| Yes | 18 | 28 | 0.394 |
| None | 28 | 31 | |
| Tumor differentiation | | | |
| III/IV | 16 | 15 | 0.297 |
| I/II | 30 | 44 | |
| TNM stage | | | |
| III/IV | 29 | 46 | 0.437 |
| I/II | 17 | 13 | |

Abbreviations: HCC, hepatocellular carcinoma; HBsAg, hepatitis B surface antigen; ALT, alanine transaminase; AFP, alpha-fetoprotein; TNM, tumor node metastasis.
* Fisher's Exact Test

**Supplementary Table S2: Univariate and multivariate analyses of factors associated with overall survival**

| Factors | Univariate, P | Multivariate | | |
|---|---|---|---|---|
| | | HR | 95%Cl | P Value |
| Sex (female vs. male) | 0.231 | | | NA |
| Age (years) (≥53 vs. <53) | 0.736 | | | NA |
| HBsAg (positive vs. negative) | 0.643 | | | NA |
| Child-Pugh classification (A vs. B) | 0.531 | | | NA |
| Liver cirrhosis (yes vs. no) | 0.327 | | | NA |
| Serum AFP, ng/mL (≥20 vs. <20) | 0.576 | | | NA |
| Serum ALT, U/L (≥75 vs. <75) | 0.634 | | | NA |
| Tumor size (diameter, cm) (≥5 vs. <5) | 0.141 | | | NA |
| Tumor number (multiple vs. single) | 0.272 | | | NA |
| Tumor differentiation (III/IV vs. I/II.) | 0.592 | | | NA |
| Tumor encapsulation (yes vs. no) | 0.085 | | | NA |
| Microvascular invasion (yes vs. no) | 0.045 | | | NS |
| TNM stage (I/II vs. III/IV) | 0.018 | | | NA |
| Annexin A2 expression (high vs. low) | 0.009 | 2.297 | 1.168-4.521 | 0.016 |

Abbreviations: 95% CI, 95% confidence interval; AFP, alpha-fetoprotein; TNM, tumor node metastasis; HBsAg, hepatitis B surface antigen; HR, hazard ratio; NA, not adopted; NS, not significant.
Cox proportional hazards regression model.

**Supplementary Table S3: Univariate and multivariate analyses of factors associated with cumulative recurrence**

| Factors | Univariate, P | Mulvariate | | |
|---|---|---|---|---|
| | | HR | 95%Cl | P Value |
| Sex (female vs. male) | 0.772 | | | NA |
| Age (years) (≥53 vs. <53) | 0.600 | | | NA |
| HBsAg (positive vs. negative) | 0.388 | | | NA |
| Child-Pugh classification (A vs. B) | 0.395 | | | NA |
| Liver cirrhosis (yes vs. no) | 0.085 | | | NA |
| Serum AFP, ng/mL (≥20 vs. <20) | 0.093 | | | NA |
| Serum ALT, U/L (≥75 vs. <75) | 0.346 | | | NA |
| Tumor size (diameter, cm) (≥5 vs. <5) | 0.163 | | | NA |
| Tumor number (multiple vs. single) | 0.134 | | | NA |
| Tumor differentiation (III/IV vs. I/II.) | 0.985 | | | NA |
| Tumor encapsulation (yes vs. no) | 0.103 | | | NA |
| Microvascular invasion (yes vs. no) | 0.048 | 1.665 | 0.944-2.936 | NS |
| TNM stage (I/II vs. III/IV) | 0.091 | | | NA |
| Annexin A2 expression (high vs. low) | 0.008 | 1.925 | 1.134-3.267 | 0.015 |

Abbreviations: 95% CI, 95% confidence interval; AFP, alpha-fetoprotein; TNM, tumor node metastasis; HBsAg, hepatitis B surface antigen; HR, hazard ratio; NA, not adopted; NS, not significant.
Cox proportional hazards regression model.

**Supplementary Table S4: Correlations between UBAP2 and Annexin A2 in 105 hepatocellular caicinoma patients**

| Variables | UBAP2 staining | | |
|---|---|---|---|
| | **high level** | **low level** | ***p* value** |
| Annexin A2 staining | | | |
| High level | 14 | 32 | **< 0.001** |
| Low level | 54 | 5 | |

**Supplementary Table S5: The target sequences of the shRNAs**

| shRNA | Target sequence |
|---|---|
| UBAP2 shRNA-1 | GAATTCTGTGGAAGAGTGG |
| UBAP2 shRNA-2 | GTACACAGCAGAATAGTAC |
| UBAP2 shRNA-3 | CATCCCAGTCCTCAGTCCT |
